# Contemporary Grand Challenges and Opportunities in Skin Allergies

**DOI:** 10.3389/falgy.2021.660447

**Published:** 2021-03-05

**Authors:** Bettina Wedi

**Affiliations:** Department of Dermatology and Allergy, Comprehensive Allergy Center, Hannover Medical School, Hannover, Germany

**Keywords:** urticaria, angioedema, atopic dermatitis, mastocytosis, drug hypersensitivity, eczema, pruritus, contact dermatitis

## Introduction to Skin Allergies

The skin is our largest organ and plays a major role in many physiological functions. No other organ demands so much attention and concern in both states of disease and health. It represents the first barrier to our environment and has fundamental importance for human interactions and mental health. In allergic skin diseases that affect a significant proportion of our population from childhood to advanced age, skin integrity is critically impaired.

It is easy to inspect the skin and to obtain biopsies. The immune system of the skin represents a complex network of keratinocytes and immune cells, including the skin-specific antigen presenting Langerhans cells. Lessons learned from allergic skin can often be transferred to other target organs, i.e., lung, nose, and gut. Accordingly, in the last decade skin allergy developed to a thriving field.

Skin allergies may start in early life, for example atopic eczema, or may occur in middle life such as urticaria, but can also develop in senescence like contact dermatitis or chronic pruritus. Some diseases are inherited such as hereditary angioedema and some are acquired by genetic alterations such as mastocytosis. Many skin allergies are long-persisting and are associated with a significant impairment of healthy related quality of life. Some skin allergies may exist only during early life such as childhood atopic dermatitis, whilst others persist for the rest of the life such as contact dermatitis. In skin allergies with periodic attacks, e.g., angioedema, the triggering factors often remain unclear. Topical treatment was used for a long time in many skin allergies, with more or less effect. Fortunately, over the past years we have considerably expanded our therapeutic armamentarium (Table 1). Moreover, additional biologic and small molecule drugs dominate pharmaceutical pipelines.

**Table 1:** Examples of skin allergy treatment approaches under investigation in clinical trials or with recent approval (in bold).

**Disease(s)**	**Drug class/target**	**Drug(s)**	**Application route**
CSU	Anti-IgE mAb	**Omalizumab**, Ligelizumab, Quilizumab^*^	s.c.
	Anti-cytokine mAb	Dupilumab (anti-IL-4/-13)	s.c.
	Anti-siglec mAb	Lirentelimab (AK002, anti-Siglec 8)	i.v.
	Receptor antagonist (RA)	Benralizumab (anti-IL5 RA), LY3454738 (anti CD200R)^*^	s.c. i.v.
	Tyrosine kinase inhibitor	Fenebrutinib (GDC0853, BTK inhibitor)^*^, Remibrutinib (LOU064, BTK inhibitor)^*^	p.o. p.o.
HAE	C1-Inhibitor	**Berinert 2000/3000**	s.c.
	Anti-Kallikrein mAb	**Lanadelumab**	s.c.
	Anti-FXII mAb	Garadacimab (CSL312)	s.c.
	Plasma kallikrein inhibitor	Berotralstat (BCX7353)	p.o.
AD	Anti-cytokine mAb	**Dupilumab (anti-IL-4/-13)**, Bermekimab (anti-IL-1α), Etokimab (anti-IL-33), Fezakinumab (Anti-IL-22), Lebrikizumab, Tralokinumab (Anti-IL-13), Nemolizumab (Anti-IL-31), Tezepelumab (Anti-thymic stromal lymphopoietin),	s.c. s.c. i.v./s.c. i.v. s.c. s.c. s.c.
	Receptor antagonist (RA)	Benralizumab (anti-IL-5 RA), RPT193 (CCR4 chemokine RA), Tradipitant (Neurokinin-1 RA), Adriforant (ZPL389, Histamine-4 RA)^*^	s.c. p.o. p.o. p.o.
	Tyrosine kinase inhibitor	**Baricitinib (JAK-1/-2-inhibitor)** Abrocitinib (PF-04965842, JAK-1 inhibitor), ASN002 (JAK/SYK inhibitor)^*^, SHR0302 (JAK-1 inhibitor),	p.o. p.o. p.o. p.o.
Cutaneous and indolent systemic mastocytosis	Receptor antagonist (RA)	Sarilumab (anti-IL-6 RA)	s.c.
	Tyrosine kinase inhibitor	Masitinib (c-Kit, Lyn and Fyn inhibitor) Avapritinib (c-Kit inhibitor)	p.o. p.o.
Prurigo nodularis	Anti-cytokine mAb	Dupilumab (anti-IL-4/-13), Nemolizumab (anti-IL-31)	s.c., s.c.,
	Receptor antagonist (RA)	Vixarelimab (KPL-716, oncostatin M receptor beta (OSMRβ), Serlopitant (neurokinin-1 RA),	s.c. p.o.
	Dual receptor antagonist (RA) and agonist	Nalbuphine (dual μ-opioid receptor antagonist and κ-agonist)	p.o.

* *Terminated because of lack of efficacy or discontinuation of further development*.

This short summary aims to describe a selection of recent achievements in the field of skin allergies, to point out knowledge gaps, and to encourage scientists to perform and communicate innovative research. It should be regarded as an invitation to all scientists and clinicians to participate open access *Frontiers in Allergy* by proposing research topics and by submitting exciting manuscripts.

## Urticaria and Angioedema: a Matter of IgE-Related Autoimmunity?

During the last years international guidelines of high quality have been established to support clinicians in the management of patients with different urticaria subtypes (1). In daily praxis, especially chronic urticaria, i.e., chronic spontaneous urticaria (CSU) and chronic inducible urticaria (CINDU), are challenging. National and international guidelines recommend the use of omalizumab, the first and only anti-IgE antibody approved for use, after failure of antihistamines at standard and increased dose (1). Nevertheless, as of now, many questions are still open regarding omalizumab in chronic urticaria. How it works is not clarified in detail, although interrupting autoimmunity type I and IIb are preferred hypotheses (2). The ligelizumab phase III trial program (Table 1) will have to confirm the promising results of the phase II trial (3) demonstrating superiority compared to omalizumab. Prospective clinical trials including CINDU subtypes and angioedema without wheals, but also including special populations, i.e., children and adolescents, elderly, obese patients, and patients with concomitant immunosuppressive or biological treatment or with cancer are required to approve efficacy and safety of omalizumab and other anti-IgE therapies (4). In addition, as of now, easy-to-use tools to identify non-responders, to predict the required duration of treatment and consented strategies on how to wean anti-IgE therapy are lacking. Additional anti-IgE approaches such as UB-221 and DARPins are under investigation (4, 5). DARPins are able to neutralize free IgE and actively dissociate pre-formed IgE:FcεRI complexes. Future insights into the mechanism of action of the various anti-IgE approaches may shed light not only on chronic urticaria and angioedema but also on other diseases in which IgE plays a major role. Whereas, until global approval of omalizumab in 2014, treatment of chronic urticaria was rather frustrating for both, patient and physician, currently, a multitude of clinical trials are on the way. Aside IgE, several other molecules not only targeting mast cells but also eosinophils, basophils, and lymphocytes are addressed (Table 1). The results will elucidate whether chronic urticaria is only a matter of IgE-related autoimmunity, or whether yet to defined urticaria endotypes must be considered that may need another specific treatment. Moreover, in urticaria pathophysiology, recently identified receptors on mast cells, such as Mas-related gene-protein coupled receptor X2 (MRGPRX2) and Siglec-8, are more and more focused (2). Interestingly enough, there is increasing evidence that these receptors are also relevant for other target cells, e.g., eosinophils and basophils (6).

## Angioedema Without Wheals: What Are the Culprit Mediators and Which Mutations Are Relevant?

The current classification of angioedema without wheals distinguishes several acquired and hereditary forms (7). In general, angioedema can be mediated by bradykinin and/or mast cell mediators including histamine (7). Nevertheless, this may be an oversimplification as increased bradykinin levels have been shown in urticaria as well (8). Both, bradykinin and histamine, are immediately degraded and therefore not available for routine assessment. Thus, biomarkers for differential diagnosis and, much more important, to predict angioedema attacks, are vitally needed. Whilst the genetic alterations of SERPING 1 gene in hereditary angioedema (HAE) resulting in C1 inhibitor deficiencies are well-known, several other genes have become into focus, for example genes for FXII, plasminogen, angiopoietin (7). In contrast, in bradykinin-mediated Angiotensin converting enzyme (ACE-)-inhibitor induced angioedema an underlying genetic predisposition has not been identified so far.

In 2019 new game changing drugs, namely subcutaneous C1-INH (Berinert 2000/3000) and Lanadelumab, got global approval for long-term prophylactic treatment of HAE (Table 1) (9). Regarding their high costs, real-world data will have to define which patients should ideally receive these drugs and at which state of their disease. Although HAE is a rare disease, several RCTs are ongoing, for example those investigating efficacy and safety of anti-FXII mAb (Garadacimab) or a plasma kallikrein inhibitor (Berotralstat) (Table 1).

## Atopic Dermatitis: Which Drug For Which Endotype?

Multiple immune pathways have been associated with atopic dermatitis and different classes of topical and systemic treatments have been developed (10). Among them are anti-cytokine biologicals, receptor antagonists, and tyrosine kinase inhibitors (Table 1). Two of them already got approval, dupilumab (anti-IL-4/-13) and baricitinib (JAK-1/-2 inhibitor). A different approach is the development of topical treatments, e.g., phosphodiesterase4 (PDE4) inhibitors such as crisaborole. Future studies will have to address which treatment is eligible for which atopic dermatitis endotype and/or in which inflammatory phase of the dermatitis. Furthermore, immunological studies might identify additional atopic dermatitis endotypes aside intrinsic and extrinsic atopic dermatitis (11). From the clinicians perspective other open questions of these new treatment approaches are long-term safety, treatment duration, and combination therapy.

## Skin Mastocytosis: How Is It Related to Systemic Mastocytosis and Allergies?

At least adult-onset skin mastocytosis is highly suggestive of systemic mastocytosis (12). So far, it has not been clarified why some patients with skin and/or systemic mastocytosis suffer from intermittent anaphylaxis and others do not. Although international guidelines give support about how to manage patients with mastocytosis, in daily practice there are many questions and few answers. Most recommendations, for example, that histamine-liberating drugs should be avoided, are based on theoretical thoughts and have not been substantiated by appropriate clinical trials. Interestingly enough, the absence of urticaria/angioedema in severe anaphylaxis might be associated with mastocytosis (13). Diagnosis and management of mast cell activation syndrome and alpha tryptasemia are debated. Very recent data identified hereditary alpha-tryptasemia as a valid genetic biomarker for severe mediator-related symptoms in mastocytosis (14). Again, in mastocytosis several new drugs such as c-kit inhibitors, receptor antibodies (Table 1), and also anti-tryptase antibodies (15) are appearing on the horizon (Table 1).

## Prurigo Nodularis, Chronic Pruritus: Can We Specify an Itch Specific Treatment?

Facing the challenges of chronic pruritus and Prurigo diseases such as prurigo nodularis is discouraging. Nevertheless, advances have been made in understanding the pathophysiology of itch (16). Regarding prurigo nodularis anti-cytokine mAbs such as dupilumab and nemolizumab (anti-IL-31), and receptor antagonists such as Vixarelimab targeting OSMRβ, which mediates signaling of interleukin-31 (IL-31) and oncostatin M (OSM) are investigated (Table 1). Moreover, for chronic pruritus other systemic treatments have been developed that target the neural system such as nalbuphine (17).

## Other Exciting Research Topics

The skin is the body's first line of defense and its frequent exposure to chemicals present in personal hygiene products, household products, or materials used in the work environment can result in the development of skin allergy. Allergic contact dermatitis is a complex skin allergy characterized by the interplay between the innate and adaptive immune system (18). There is increasing evidence that allergic contact dermatitis could be treated by biologics but prospective clinical trials are lacking (19).

Another interesting question is the role of the skin microbiome, not only in relation to the body (20) but also related to the development of (skin) allergies (21).

Finally, most drug hypersensitivity reactions manifest on the skin (22). The use of biologics, immune check point inhibitors and small molecule drugs constantly increases our understanding of the complex skin immune system. The border between adverse drug reactions and drug hypersensitivity is floating and for most reactions the pathomechanism has not been clarified so far (23).

## Conclusion and Perspectives

**Figure 1:**
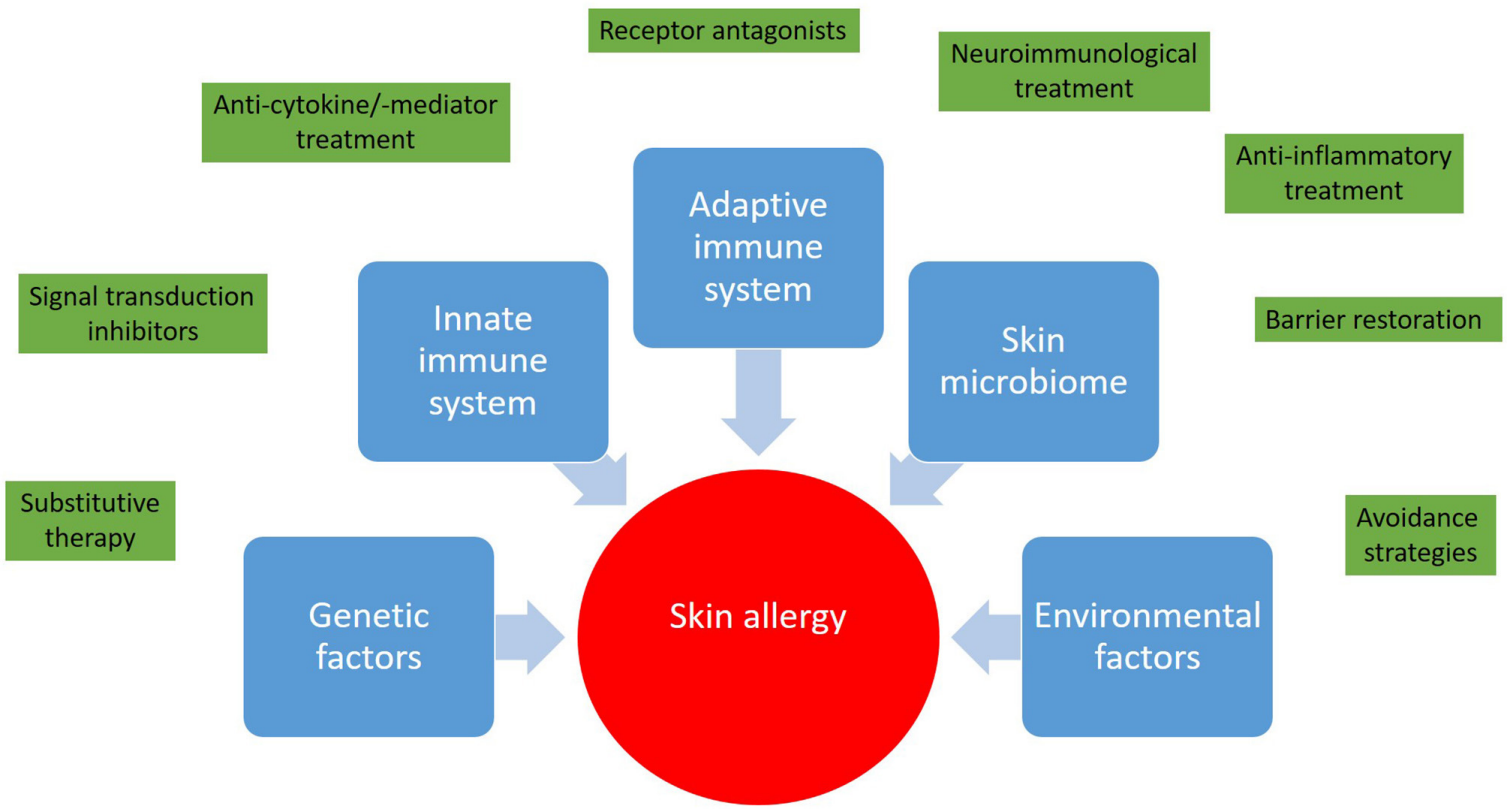
Skin allergies result from a deregulated interplay between genetic predisposition, the innate and adaptive immune response, and environmental factors. In light of this complexity a wide range of therapeutic approaches from avoidance strategies to targeted therapies and immunotherapies has been developed.

It is great to see that with regard to skin allergies the number of clinical trials is increasing. The outcome of innovative treatment approaches will expand our horizons and deepen our understanding of skin immunology and skin allergy (Figure 1). Interestingly enough, albeit the itch in CSU, in atopic dermatitis, and prurigo nodularis is considered to be different from a pathophysiologic point of view, some treatment approaches, for example dupilumab (anti-IL-4/-13), are investigated in all these diseases. Growing knowledge might enable step-by-step adjustment of our diagnostic and treatment algorithms toward precision medicine (24). Nevertheless, at the moment each milestone opens new questions. The skin is easy to access, therefore skin allergies are attractive for research purposes and hopefully young scientists and clinicians will be attracted to solve remaining and upcoming mysteries with the goal of prescribing the right medication, at the right dose, to the right patient.

## Author Contributions

The author confirms being the sole contributor of this work and has approved it for publication.

## Conflict of Interest

The author declares that the research was conducted in the absence of any commercial or financial relationships that could be construed as a potential conflict of interest.
